# Incidence of SARS-CoV-2 Infection in Health Care Workers After a Single Dose of mRNA-1273 Vaccine

**DOI:** 10.1001/jamanetworkopen.2021.16416

**Published:** 2021-06-16

**Authors:** Kalpana Gupta, William J. O’Brien, Pamela Bellino, Katherine Linsenmeyer, Sucheta J. Doshi, Robert S. Sprague, Michael E. Charness

**Affiliations:** 1 VA Boston Healthcare System, Boston, Massachusetts

## Abstract

This cohort study of health care workers in a metropolitan Veterans Administration network examines the association of SARS-CoV-2 infection after receiving a single dose of mRNA-1273 vaccine compared with no vaccination.

## Introduction

A 2021 randomized clinical trial^1^ demonstrated remarkably high efficacy of mRNA-1273 vaccine in reducing symptomatic SARS-CoV-2 infection after 2 doses administered 28 days apart; however, low numbers of infection during the first 2 weeks following dose 1 prevented full assessment of early vaccine efficacy. VA Boston Healthcare System (VABHS) began vaccinating health care workers (HCWs) during an early winter surge in SARS-CoV-2 infections in Massachusetts.^2^ A concomitant large increase in SARS-CoV-2 infections in our workforce provided an opportunity to evaluate the association between receipt of dose 1 of mRNA-1273 vaccine and SARS-CoV-2 infection.

## Methods

For this cohort study, we performed a retrospective survival analysis using a Cox proportional hazards model with a single time-varying treatment to estimate the hazard ratio (HR) of acquiring SARS-CoV-2 in vaccinated compared with unvaccinated HCWs. All VABHS clinical and nonclinical HCWs were included, and the study period was 42 days starting on December 22, 2020, the first day of vaccine availability. Treatment status changed from unvaccinated to vaccinated on the date of dose 1, and time prior to vaccination contributed to unvaccinated risk. Dose 2 was administered 28 days after dose 1 (or within 4 days before or after that date). The primary end point was a SARS-CoV-2 reverse transcription–polymerase chain reaction (RT-PCR) positive test.^3^ All HCWs were offered mRNA-1273 vaccine in accord with US Centers for Disease Control and Prevention priority guidelines. Testing was triggered by policy for HCWs with symptoms, exposure, or for mandatory surveillance on selected units.^3^ To account for the time required for the development of immunity,^4^ we calculated 2 additional HRs excluding infections arising in the vaccinated group before days 8 and 15 following dose 1, respectively. Vaccine effectiveness was calculated as 100% × (1−HR). Analyses were performed in R version 4.0.4 (R Project for Statistical Computing). *P* < .05 was considered significant in 2-sided tests. This study followed Strengthening the Reporting of Observational Studies in Epidemiology (STROBE) reporting guideline for cohort studies. Race was self-classified in human resources databases and was included for analysis because of racial differences in vaccine hesitancy and community transmission. Because risk to participants was minimal and the study was deemed a quality improvement project, informed consent and review were exempted by the VABHS institutional review board.

## Results

The cohort comprised 4028 total HCWs (mean [SD] age, 48.1 [12.1] years; 2473 [61.4%] women; 2682 [66.6%] White, 810 [20.1%] Black) (Table), of which 3367 (83.6%) were vaccinated during the study period. SARS-CoV-2 infection was identified by RT-PCR in 107 HCWs: 39 vaccinated and 68 unvaccinated. Most infections (75 infections [73.5%]) were symptomatic, and 7 (6.5%) were identified during routine surveillance (Table). Vaccinated and unvaccinated RT-PCR–positive HCWs did not differ significantly by age, gender, race, proportion of nursing staff, or clinical presentation. Among the 39 SARS-COV-2–positive vaccinated HCWs, 26 (66.7%) received dose 1 before December 29, 2020. Vaccine clinical effectiveness was 50.3% (95% CI, 23.0%-67.9%) for the entire 42-day period of follow-up, 77.5% (95% CI, 61.2%-87.0%) for days 8 through 42, and 95.0% (95% CI, 86.0%-98.2%) for days 15 through 42 (Figure). Clinical effectiveness was similar when analysis was limited to the initial 28 days of study, thereby excluding dose 2 effects.

**Table.** Demographic and Clinical Characteristics of SARS-CoV-2–Positive HCWs

Characteristics	HCWs, No. (%)			*P* value^**a**^
	Total (n = 4028)	SARS-CoV-2 positive		
		Vaccinated (n = 39)	Unvaccinated (n = 68)	
Demographic^b^				
Age, mean (SD), y	48.1 (12.1)	47.9 (17.0)	42.5 (13.2)	.07
Female	2473 (61.4)	21 (53.8)	45 (66.2)	.21
Male	1555 (38.6)	18 (46.2)	23 (33.8)	
Race^c^				
Black	810 (20.1)	4 (10.3)	14 (20.6)	.14
White	2682 (66.6)	29 (74.4)	50 (73.5)	
Other	492 (12)	6 (15.4)	4 (5.9)	
Nursing staff	1139 (28.3)	15 (38.5)	23 (33.8)	.63
Indication for testing				
Symptoms	NA	25 (64.1)	50 (73.5)	.42
Exposure	NA	10 (25.6)	15 (22.1)	
Surveillance^d^	NA	4 (10.3)	3 (4.4)	
Clinical condition at time of positive PCR				
Symptomatic	NA	27 (69.2)	51 (75.0)	.68
Presymptomatic	NA	4 (10.3)	4 (5.9)	
Asymptomatic	NA	8 (20.5)	13 (11.8)	

Abbreviations: HCW, health care worker; NA, not applicable.

^a^ *P* values represent comparisons of vaccinated and unvaccinated SARS-CoV-2–positive HCWs. Age was compared using a *t *test, and percentages were compared using χ^2^*.*

^b^ Demographic data were approximated for the entire population based on human resources databases but were not stratified for vaccination status.

^c^ Race was self-identified from administratively defined options. Other includes Asian, Native Hawaiian or Pacific Islander, American Indian or Alaska Native, and 2 or more races.

^d^ At least weekly surveillance was conducted on an average of 400 HCWs during the study period.

**Figure.**
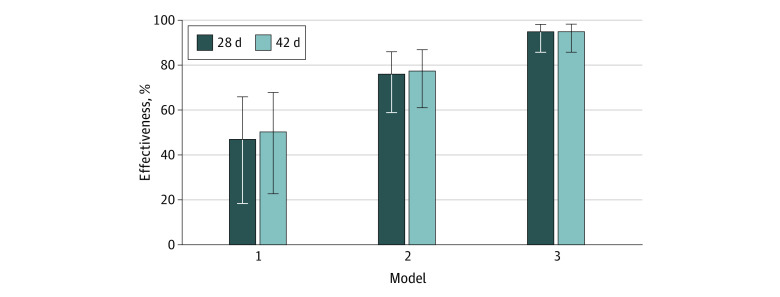
Clinical Effectiveness of Dose 1 of mRNA-1273 Vaccine Against SARS-CoV-2 Infection Hazard ratios were computed for all health care workers from day 1 (model 1), day 8 (model 2), or day 15 (model 3) after receipt of dose 1 until either day 28 (dark bars) or day 42 (light bars). Error bars indicate 95% CIs. Limiting the analysis to the period before dose 2 (28 days) did not change the outcome.

## Discussion

This study demonstrated an association between receipt of mRNA-1273 vaccine and a reduction in SARS-CoV-2 infection in HCWs beginning 8 days after dose 1. These real-world findings reflect vaccination solely with mRNA-1273 and are consistent with aggregated data for BNT162b2 and mRNA-1273 in HCWs.^4,5,6^ The first-dose risk reduction of 95% after day 14 highlights the potential for vaccination with mRNA-1273 to rapidly mitigate surges of vaccine-sensitive SARS-CoV-2 infection in HCWs.

Limitations of the study include the observational design, the small number of participants from a single center, and the inability to adjust for confounding characteristics; hence, results may not generalize to other settings with different demographic characteristics. The period of risk for infection was slightly later for the vaccinated cohort than the unvaccinated cohort. However, the study encompassed a sustained period of high community transmission.^2^ Black HCWs constituted a higher percentage of the SARS-CoV-2–positive unvaccinated cohort, although these data were not statistically significant. Finally, these findings address primarily the short-term effects of a single dose of mRNA-1273 vaccine.
